# Mimicry in Cutaneous Malignancy—Rare Forms of Mycosis Fungoides as Diagnostic Pitfalls: A Narrative Review

**DOI:** 10.3390/medicina62040616

**Published:** 2026-03-24

**Authors:** Marija Malinić, Branislav Lekić, Dubravka Živanović

**Affiliations:** 1 Clinic of Dermatology and Venereology, University Clinical Center of Serbia, 11000 Belgrade, Serbia; marija.malinic@gmail.com (M.M.); branislav.lekic@med.bg.ac.rs (B.L.); 2 Faculty of Medicine, University of Belgrade, 11000 Belgrade, Serbia

**Keywords:** cutaneous T-cell lymphoma, mycosis fungoides, bullous mycosis fungoides, icthyosiform mycosis fungoides, folliculotropic mycosis fungoides, hypopigmented mycosis fungoides, poikilodermatous mycosis fungoides, granulomatous slack skin, pagetoid reticulosis, syringotropic mycosis fungoides

## Abstract

Mycosis fungoides (MF) is a rare primary cutaneous T-cell lymphoma (pCTCL) that generally has an indolent course with a favorable prognosis. However, numerous clinical variants have been described that differ substantially from classic Alibert–Bazin MF, resulting in altered prognosis, treatment response, and patient outcomes. This narrative review considers rare MF variants—bullous, ichthyosiform, hypopigmented, folliculotropic, poikilodermatous, granulomatous, granulomatous slack skin, pagetoid reticulosis and syringotropic MF—with emphasis on practical diagnostic approaches for clinicians. Given that MF can mimic more than 50 different dermatoses and is frequently associated with prolonged diagnostic delay, we provided detailed clinical and dermoscopic features that should raise diagnostic suspicion and guide biopsy decisions. We discussed extensive differential diagnoses for each variant and highlighted MF’s status as dermatology’s “great imitator.” Additionally, we addressed the risk of second primary malignancy in patients with MF, as well as the genetic and microenvironmental factors proposed to contribute to its clinical heterogeneity. Furthermore, we evaluated existing classification systems and suggested future directions that integrate molecular data and tumor biology to improve prognostic assessment and guide therapeutic decision-making.

## 1. Introduction

Primary cutaneous lymphomas are a heterogeneous group of T- and B-cell lymphomas that affect the skin with no evidence of extracutaneous disease at the time of diagnosis [1]. Mycosis fungoides (MF) represents the most common form of primary cutaneous T-cell lymphoma (pCTCL) and accounts for about 60% of pCTCL [1]. French physician Jean Louis Alibert first described MF in 1806, documenting its characteristic mushroom-like skin tumors [2]. Bazin’s subsequent definition of the three-stage progression (patch, plaque, tumor) in 1870 established a framework that remains central to understanding disease progression, with both contributions recognized in the eponym “Alibert–Bazin”, which now refers to the classic MF presentation [3,4].

About 70–75% of MF patients present with the classical form, which is characterized by persistent, ill-defined, progressive erythematous patches and/or plaques of variable size and shape localized on sun-protected skin [5]. The histopathologic (HP) hallmark of MF is epidermotropism of small- to medium-sized lymphocytes with immunohistochemical (IHC) analysis demonstrating that these cells predominantly display a CD4+ T-helper phenotype [6]. Classic MF (CMF) has a male predominance, with a median age of diagnosis being in the mid-50s and 60s [7,8]. Pediatric and adolescent MF is a rare entity, with the hypopigmented variant representing the most common subtype and a median age at diagnosis of approximately 12 years [9]. Although the documented incidence of MF ranges from 0.2 to 0.6 new cases per 100,000 persons per year, the actual disease burden is likely far greater, as diagnostic delays and frequent misdiagnosis result in significant underreporting [10]. A study by Ottevanger et al. (2021) demonstrated a 2.42-fold increase in the incidence of MF and Sézary syndrome (SS) in the Netherlands over the past two decades [11].

Current evidence indicates that MF exhibits remarkable clinical variability due to a complex interplay of biological, molecular, and microenvironmental factors that fundamentally shape disease presentation and behavior [12,13]. This heterogeneity extends far beyond simple morphologic variation, reflecting the disease’s underlying complexity at multiple levels.

This narrative review addresses rare forms of MF, with the primary aim of equipping clinicians with practical tools for earlier recognition. By detailing the clinical presentations, dermoscopic patterns, and differential diagnoses of rare MF subtypes, we aim to lower the threshold for diagnostic suspicion and biopsy in cases that deviate from typical inflammatory patterns. Early recognition of these MF variants can fundamentally alter patient outcomes through timely, stage-appropriate intervention.

## 2. Bullous Mycosis Fungoides

Bullous mycosis fungoides (BMF) is a rare variant of MF, initially described by Moritz Kaposi in 1887 as pemphigus-like mycosis fungoides [14]. To date, approximately 40 cases have been documented in the literature [15]. Although the rarity of the condition limits epidemiological analysis, existing literature shows a slight predilection for male patients, with reported cases spanning ages from 30 to 90 years old, with a predominance in the seventh and eighth decade of life [16,17].

**Figure 1:**
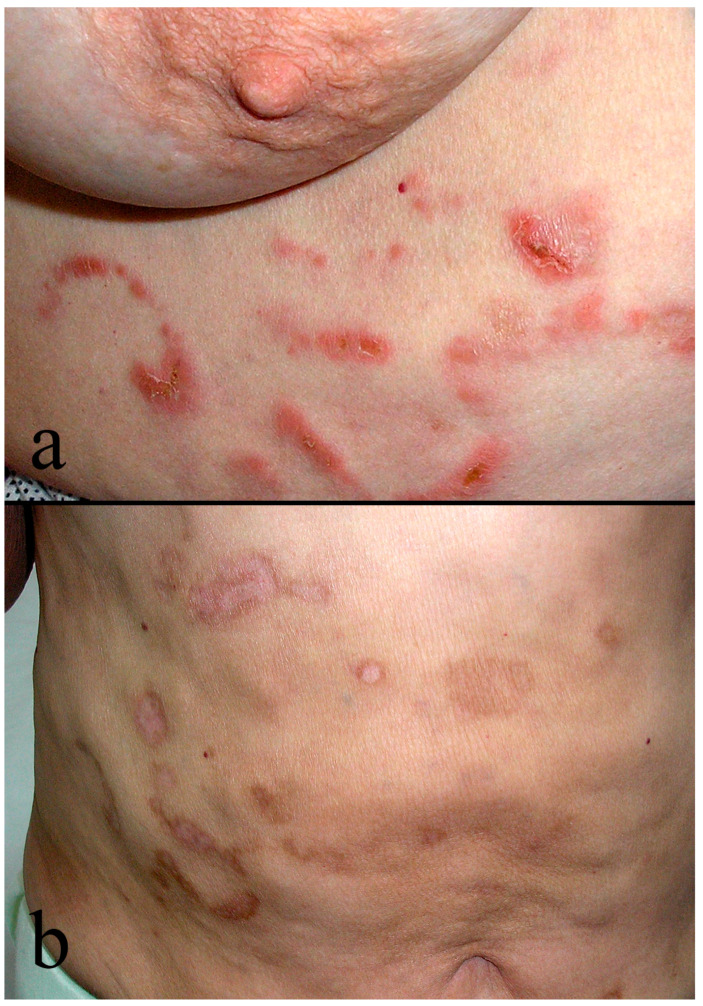
Clinical features of bullous mycosis fungoides (BMF). (**a**) Annular erythematous plaques with superficial erosions on the trunk. (**b**) Residual atrophic cicatrices following resolution of bullous lesions.

Clinically, the disease is characterized by flaccid or tense vesiculobullous lesions, most often appearing alongside patches, plaques, and tumors (Figure 1a,b). The lesions can be localized or disseminated, predominantly on the trunk and upper and lower extremities, on healthy skin or within MF patches and plaques [14,18]. Data on subjective symptoms in BMF remain limited; pain may result from erosions and ulcerations, whereas pruritus frequently persists due to the underlying condition [16,17]. Proposed mechanisms of bullae formation include confluence of Pautrier’s microabscesses or loss of keratinocyte cohesion due to proliferation of atypical lymphocytes [15]. Typically, bullae appear months to years after the onset of classic patches and plaques and occasionally may present as the initial manifestation [14,19]. The vesiculobullous phenotype of MF has a more aggressive clinical course that typically develops in patients with pre-existing, often advanced-stage disease, which may account for its predominance among older individuals with longer disease duration [16]. Bullous lesions are generally considered an unfavorable prognostic sign and are associated with rapid clinical progression [16,18].

This clinical behavior may be driven by a T-helper type 2 (Th2)-dominant cytokine milieu characteristic of advanced disease. Supporting this, Momtahen et al. demonstrated elevated interleukin-5 (IL-5) and interleukin-10 (IL-10) expression in atypical lymphocytes compared to interferon-γ (IFN-γ), suggesting that the vesicular and eczematous features, along with the aggressive course, may reflect Th2 immune polarization [19].

The vesiculobullous presentation may lead to an initial misdiagnosis as a reactive dermatosis. Multiple biopsies and expert dermatopathology reviews are often necessary. The overlap with inflammatory conditions (particularly those with spongiosis) creates diagnostic pitfalls [20]. Vesiculobullous skin lesions have a broad differential diagnosis encompassing multiple etiologic categories and require systematic evaluation. The differential diagnosis must exclude autoimmune bullous diseases (pemphigus variants, bullous pemphigoid, linear IgA dermatosis, etc.), acute contact dermatitis, infectious diseases (impetigo, staphylococcal scalded skin syndrome, herpes simplex virus, varicella-zoster virus, and fungal infections), reaction to arthropod stings, thermal injuries (burns), drug-related bullous eruptions (Stevens–Johnson syndrome, toxic epidermal necrolysis), genetic disorders (inherited epidermolysis bullosa variants, Hailey-Hailey disease, Darier disease), metabolic conditions (*porphyria cutanea tarda*), and even *dermatitis artefacta* [14,17,21,22]. Also, the coexistence of two different clinical entities should be considered. The literature documents several cases of MF occurring concurrently with autoimmune bullous diseases, including both *pemphigus foliaceus* and bullous pemphigoid [23,24].

In 2001 Bowman proposed four defining criteria for the diagnosis of BMF: (1) clinically apparent vesiculobullous lesions with or without typical MF lesions, (2) typical HP features of MF with intraepidermal or subepidermal blisters, (3) negative immunofluorescence to exclude autoimmune bullous diseases, and (4) negative evaluation for other causes of vesiculobullous lesions (medications, infection, porphyria, and phototherapy) [17]. This remains the standard for diagnosing BMF and emphasizes the need to exclude other vesiculobullous etiologies [17].

Direct and indirect immunofluorescent tests are negative. HP analysis of BMF is characterized by intraepidermal or subepidermal blister formation, along with typical MF findings [25]. The immunophenotype of BMF closely resembles that of CMF and is characterized by CD3 and CD4 positivity, loss of CD5 and CD7 expression, absence of CD8, variable CD30 expression, and typically negative cytotoxic markers, including TIA-1, granzyme B, and perforin [15].

Therefore, accurate diagnosis requires integrating patient age, medical history, medication use, lesion distribution and morphology, mucosal involvement, HP evaluation, direct and indirect immunofluorescence, serologic testing, and specific laboratory findings.

## 3. Ichthyosiform Mycosis Fungoides

Ichthyosiform mycosis fungoides (IMF) is a rare variant of cutaneous T-cell lymphoma that clinically manifests as dry, scaling patches and plaques mimicking acquired ichthyosis, with HP features of MF and ichthyosis vulgaris [26]. According to the available literature, this form of MF was first described in the late 20th century, and most authors attribute the initial description of this variant to Kütting et al., who reported a 25-year-old man with an ichthyosis-like eruption of the trunk, back in 1996 [27]. Over time, IMF was accepted by multiple authors as a distinct variant within the spectrum of MF [28,29,30,31,32]. Based on data collected over approximately 30 years, Marzano et al. estimated the prevalence of IMF to be 1.8% [28].

**Figure 2:**
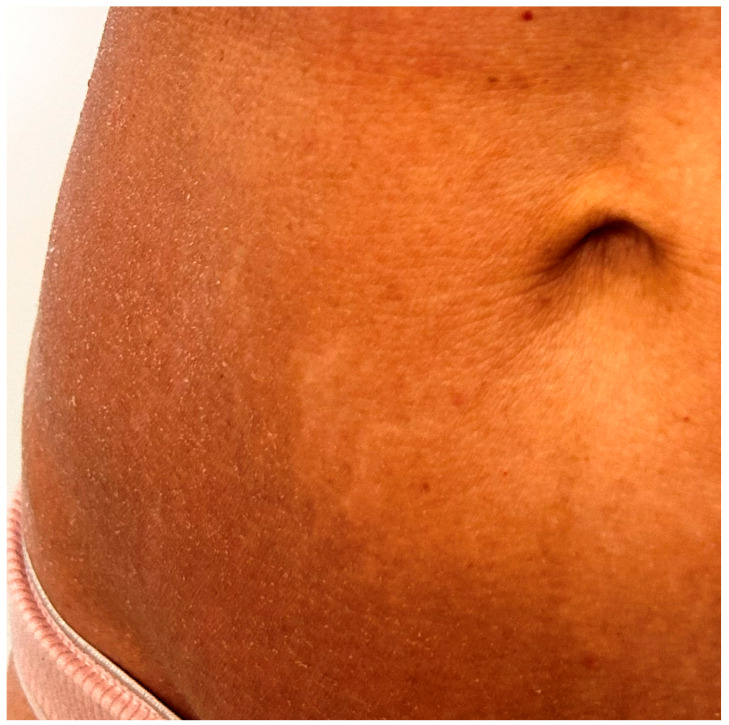
Clinical features of ichthyosiform mycosis fungoides (IMF). Patches and plaques with fine white scale involving the trunk.

The lesions typically manifest as fine, whitish scaling patches resembling ichthyosis vulgaris, often affecting the trunk and extremities (Figure 2) [33]. Pruritus is prominent, and excoriations may be a part of the clinical presentation [31]. The ichthyosiform eruption may occur as an isolated manifestation of MF, but more frequently appears in combination with conventional MF patches or plaques, folliculotropic lesions, or other variants such as hypopigmented and poikilodermatous MF [29]. This type of MF usually has an indolent course, responds well to skin-directed therapies (SDTs), and has a favorable outcome. However, when associated with folliculotropism, the disease may show greater resistance to topical treatments and require more aggressive skin-directed approaches or systemic therapy [34].

Acquired ichthyosis (AI) represents the key differential diagnosis of IMF. Its recognition requires careful distinction between benign secondary causes and an underlying malignant etiology. AI has been described in association with a broad spectrum of systemic conditions, including autoimmune disorders such as Sjögren’s syndrome, endocrine abnormalities such as hypothyroidism, metabolic disturbances caused by malnutrition, exposure to certain medications (statins, allopurinol, antipsychotics, etc.), and chronic renal failure [35,36]. In cases of generalized ichthyosiform eruption, particular attention should be paid to the exclusion of underlying malignancy, especially lymphoproliferative neoplasms such as Hodgkin’s lymphoma, anaplastic large cell lymphoma, and adult T-cell leukemia/lymphoma (ATLL), for which testing for human T-cell leukemia virus type 1 (HTLV-1) is recommended [29,33,37]. Other differential diagnoses include atopic dermatitis with lichenification and xerosis, particularly in pediatric patients, where hypopigmented and folliculotropic MF variants are common, as well as chronic eczema and even psoriasis with prominent scaling, for which dermoscopic features can sometimes help distinguish it from MF [34,38].

IMF shares the fundamental HP features of CMF, but with additional epidermal changes characteristic of ichthyosis. The key HP distinction is that the IMF, besides epidermotropic infiltration of atypical lymphocytes, shows compact hyperorthokeratosis, a thinned or absent granular layer, and acanthosis [39]. IMF typically demonstrates a CD2+/CD3+/CD4+/CD5+/CD8− immunophenotype with frequent loss of CD7 and negative cytotoxic markers. However, some cases show a CD8+ cytotoxic variant (CD3+/CD4−/CD8+/TIA1+) and a rare subset exhibits a CD4/CD8 double-negative phenotype (CD3+/CD4−/CD8−) with loss of CD7 [40].

Clinical awareness of IMF, a rare entity, is essential for the evaluation of adult-onset ichthyosiform eruptions, as misclassification as AI may delay diagnosis of MF. Unlike paraneoplastic AI, which may signal aggressive systemic lymphoma or other underlying malignancy, IMF is typically an early-stage, skin-limited disease with an indolent course and favorable response to SDTs. Recognition of concomitant folliculotropic involvement is important, as it may influence therapeutic strategy and require closer monitoring.

## 4. Hypopigmented Mycosis Fungoides

Hypopigmented mycosis fungoides (HMF) is a rare clinical variant of MF characterized by hypopigmented to achromic patches and plaques, predominantly localized on the proximal portions of the extremities and the trunk in a bathing-suit pattern, especially affecting the gluteal region [41,42]. According to available literature, the first case of primary HMF was described by Ryan et al. in 1973 [43]. HMF predominantly affects children, adolescents, and young adults, shows a strong predilection for darker skin phototypes (Fitzpatrick skin type IV–VI), and has female predominance [41,44,45].

**Figure 3:**
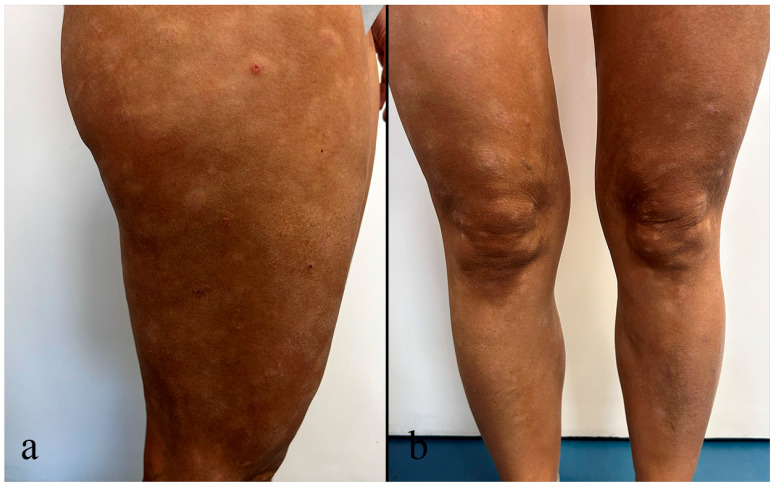
(**a**,**b**) Clinical features of hypopigmented mycosis fungoides (HMF). Ill-defined, hypopigmented, disseminated patches and plaques affecting the lower extremities.

The number, size, and shape of the lesions are variable; sometimes they are poorly circumscribed and of irregular shape (Figure 3a,b), affecting a large body surface area, even the face and neck [46]. Lesions are occasionally accompanied by pruritus, telangiectasia, and/or atrophy [41]. Combined presentations are common, particularly the combination of hypopigmented and folliculotropic MF [9]. HMF has an indolent course and carries exceptional prognostic significance because it represents one of the most favorable MF variants, especially when diagnosed in children [44,47].

The differential diagnosis of HMF is extensive and challenging, as the clinical presentation closely resembles numerous benign hypopigmented dermatoses. However, HMF has several clinical mimickers that require careful differentiation. One of them is vitiligo, characterized by complete depigmentation, well-defined borders, absence of scaling or erythema, and negative HP for lymphocytic infiltration. Dermoscopy of a vitiligo lesion reveals a starburst pattern, micro-koebnerization, and trichrome pattern [48]. Pityriasis alba, commonly seen in children, presents with hypopigmented patches and fine scaling, lacks epidermotropic lymphocytes, and resolves spontaneously. Post-inflammatory hypopigmentation is differentiated by a history of preceding inflammation and spontaneous repigmentation [42]. Other conditions, such as idiopathic guttate hypomelanosis, hypopigmented pityriasis versicolor, *nevus depigmentosus*, lichen sclerosus, tuberous sclerosis (ash leaf macules), and pityriasis lichenoides chronica (PLC), can also present with hypopigmented macules but have distinct HP and clinical features [42,49,50]. Lastly, inflammatory dermatoses such as atopic dermatitis and psoriasis can mimic early-stage HMF, and dermoscopy plays an important role in differentiating these conditions based on vascular patterns and scaling characteristics. Dermoscopy of HMF shows white to pink structureless areas with patchy whitish scales often along skin furrows and in the perifollicular area. Contrary to psoriasis and atopic dermatitis, the blood vessel density in MF is lower and exhibits a linear and spermatozoa-like pattern [38]. In psoriatic lesions, the dotted vessels pattern is uniformly distributed against a bright-red background with diffuse scaling [38]. In contrast, the scale associated with atopic dermatitis has a yellowish-orange appearance [38].

On HP examination, HMF is characterized by less pronounced dermal infiltration, reduced dermal atypia, and a lower frequency of Pautrier microabscesses, while maintaining equal intensity of epidermotropism [45]. HMF exhibits a markedly distinct immunophenotypic profile compared with CMF, with some studies reporting a CD3+/CD4−/CD8+ cytotoxic phenotype in up to 95% of cases [45]. IHC staining with Melan-A and HMB-45 can help differentiate HMF, which shows reduced but detectable melanocytes, from vitiligo, in which melanocytes are completely absent [42].

The diagnosis of HMF is often challenging because of its close resemblance to common benign hypopigmented dermatoses. Despite its generally favorable prognosis, the clinical burden and implications of a chronic lymphoproliferative disorder should not be underestimated. In this context, dermoscopy is a valuable tool in everyday dermatologic practice, complementing HP for improving lesion assessment and biopsy selection.

## 5. Folliculotropic Mycosis Fungoides

Folliculotropic mycosis fungoides (FMF) is a distinct variant of MF characterized by infiltration of neoplastic T-cells into hair follicles, and the first description of this particular variant of MF dates to the 1960s [51]. Since the early 2000s, FMF has been recognized as a distinct disease entity in the World Health Organization—European Organization on Research and Treatment of Cancer (WHO-EORTC) classification of primary cutaneous lymphomas [52]. According to the literature, FMF shows a slight male predilection and accounts for approximately 12–17% of all MF cases in adult cohorts and represents the second most common clinical variant in pediatric patients, following HMF [9,53,54]. Pruritus is typically intense and represents a significant clinical burden in this MF variant [55]. Wieser et al. reported that approximately one-third of patients in their FMF cohort progressed to advanced-stage disease and concluded that, overall, FMF is associated with a worse overall 10-year survival rate compared with other MF variants [56].

**Figure 4:**
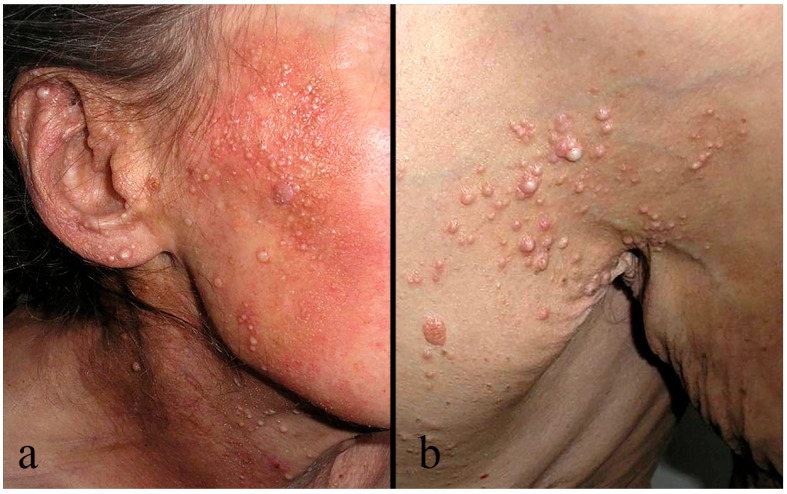
(**a**,**b**) Clinical features of folliculotropic mycosis fungoides (FMF). Multiple cysts, papules, and comedones involving the right cheek and earlobe, neck, and left periaxillar region, accompanied by marked erythema on the face and neck.

To evaluate disease stage, many authors suggest a paradigm shift beyond the traditional TNMB classification [56,57]. This distinction requires integration of clinical morphology and HP features [56]. FMF presents as two distinct clinicopathologic subtypes with fundamentally different prognostic implications and treatment responses; therefore, the distinction between early- and advanced-stage disease can be made [51,54,55,56,57]. If distinctions cannot be made on clinical grounds alone, HP evaluation plays an important, complementary role in determining the stage of FMF. Early stage FMF presents with follicle-based scaly papules, patches, and/or thin plaques, keratosis pilaris-like lesions, and/or acneiform lesions on the trunk and extremities [54,58]. In addition, comedo-like plugs and epidermal cysts, predominantly affecting the head and neck region, can also be seen in the early stage of disease, though this anatomic distribution differs from the typical early-stage presentation (Figure 4a,b) [57,58,59]. In advanced/tumor stage disease, FMF is defined clinically by infiltrated thick plaques and/or tumors with a preference for the head and neck region; alopecia and eyebrow involvement are often featured [57,58]. Due to hair follicle destruction, hair growth can be affected in all hair-bearing regions. Reports indicate that alopecia in FMF patients ranges from 33 to 78% and is more common in advanced stages [56]. Overlap in clinical presentation may occur, with some patients exhibiting features of both HMF and FMF [9].

Dermoscopic findings in FMF include perifollicular accentuation, defined as a whitish halo or ring-like structure around the hair follicle opening. Although dermoscopic features of MF are largely non-specific and should be interpreted in conjunction with clinical and histopathologic findings, perifollicular accentuation and spermatozoa-like vessels represent relatively specific dermoscopic clues that may support the diagnosis [38].

Given the clinical manifestations of FMF, the differential diagnosis spans numerous inflammatory, infectious, and granulomatous dermatoses, as well as follicular disorders. FMF can mimic various forms of acne with open and closed comedones, inflammatory papules, pustules, and nodules, as well as acneiform eruptions. Hidradenitis suppurativa, keratosis pilaris, folliculitis, furunculosis, rosacea, lichen planopilaris, and follicular mucinosis, in some aspect, share clinical resemblance to FMF [60]. Granulomatous conditions, including sarcoidosis and cutaneous leishmaniasis, should also be excluded in the differential diagnosis [61]. Follicular involvement leading to patchy alopecia when assessed as an isolated manifestation can be misleading; therefore, a comprehensive full-skin examination is essential, regardless of the patient’s primary presenting complaint [62].

HP analysis demonstrates infiltration of the follicular epithelium by atypical lymphocytes (folliculotropism), commonly accompanied by mucinous degeneration of the follicular epithelium (follicular mucinosis) [55,59,63,64]. Eosinophils are consistently observed in cases characterized by mucin deposition and may, in certain instances, represent a prominent component of the inflammatory infiltrate [55]. The immunophenotype is similar to CMF, with a CD3+/CD4+/CD8−/CD7− profile and frequently elevated CD4:CD8 ratios (≥4:1 to 10:1) [65]. CD8+ cases are exceptionally rare [65].

Distinguishing between chronic, inflammatory, benign conditions and this MF variant, which can have a poor prognosis in advanced-stage disease, is highly significant to start adequate treatment promptly. Recurrence, presence or absence of a progressive course, personal and family history, distinct lesion distribution, and complete clinical evaluation, including inflammoscopy, can help guide further diagnostic protocols.

## 6. Poikilodermatous Mycosis Fungoides

Poikilodermatous mycosis fungoides (PMF) is a distinct clinical variant of MF characterized by the triad of atrophy, telangiectasia, and mottled pigmentation, typically in flexural areas or with generalized distribution [31,66]. By the late 20th century, PMF was established as a clinicopathological entity distinct from CMF [31,67]. Unlike the CMF, in some studies, PFM demonstrates a relative female predominance and almost always has a chronic, indolent course and a favorable prognosis [53,66,68]. Sidiropoulou et al. reported that PFM occurred in 5.5% of a cohort of 688 MF cases and was associated with a pronounced diagnostic delay, averaging 12.5 years, underscoring its role as a significant diagnostic pitfall [53].

**Figure 5:**
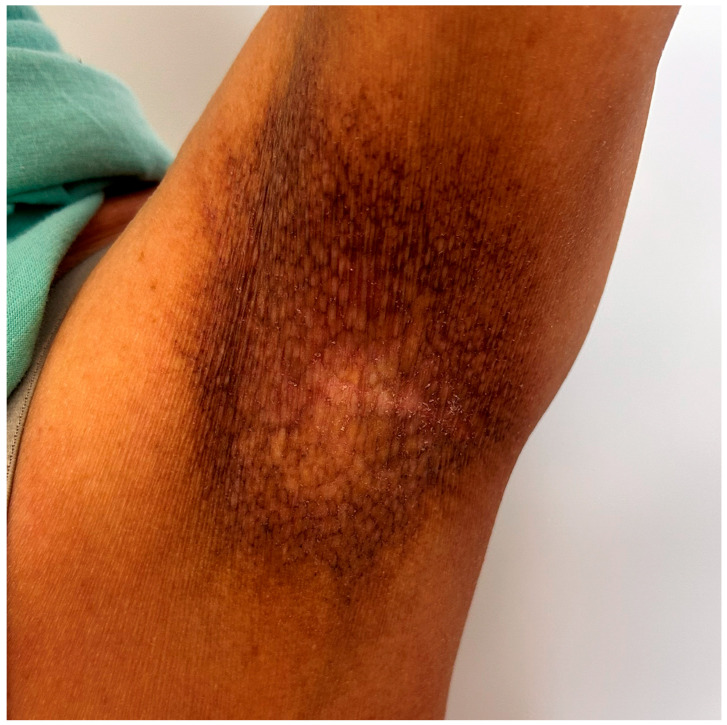
Clinical features of poikilodermatous mycosis fungoides (PMF). Hallmark triad—atrophy, telangiectasia, hyperpigmented and hypopigmented macules—with characteristic reticulated appearance affecting the left axillar region.

The previously mentioned hallmark triad—atrophy, telangiectasia, and mottled pigmentation—produces a distinctive reticulated or “salt and pepper” appearance. Clinically, atrophy manifests as thinning of the skin, telangiectasia as fine superficial vessels, and pigmentary changes as a mixture of hyperpigmented and hypopigmented macules (Figure 5) [69,70]. Lesions may be annular or irregular in shape, and their size and configuration can vary over time. Scaling is usually fine and less prominent than in psoriasis or eczema [38]. Dermoscopy can assist and reveal multiple whitish and brown, structureless, polygonal areas, patchy scales, and dotted or hairpin-like blood vessels [38,71]. In patients with skin of color, diagnosing PMF can be challenging because clinical features can be misleading. Hyperpigmentation tends to be more pronounced, and hypopigmented macules may be less prominent or may blend with the surrounding skin, making the classic “mottled” appearance of poikiloderma less obvious [72].

The differential diagnosis is broad and includes inflammatory dermatoses such as psoriasis, connective tissue diseases including dermatomyositis and lupus erythematosus, pigmentary disorders such as lichen planus pigmentosus, and other forms of cutaneous lymphoma, reflecting the considerable clinical and HP overlap among these conditions. The main distinction should be made towards dermatomyositis. Poikiloderma in dermatomyositis typically occurs in photoexposed regions affecting the neck and upper chest (V-sign), posterior shoulders and upper back (shawl sign), and extensor surfaces of the arms, whereas PMF favors sun-protected areas of skin [73]. The lack of pathognomonic cutaneous signs of dermatomyositis (heliotrope rash, Gottron’s papules, and Gottron’s sign), the absence of muscle involvement and systemic symptoms, when combined with specific anamnestic data (indolent course, lack of photosensitivity) and the distribution of skin lesions (sun-protected areas), should favor a PMF diagnosis over dermatomyositis [73]. Discoid lupus erythematosus (DLE) can be clinically distinguished from PMF by well-demarcated, erythematous plaques with adherent scale, central atrophy, follicular plugging and photosensitive distribution [74]. Lichen planus pigmentosus and post-inflammatory hyperpigmentation are important mimickers in patients with skin of color and must be excluded in the differential diagnosis of PMF [72].

Conditions that may partially resemble PMF include PLC, which follows a waxing and waning course, and benign pigmented purpuric dermatosis (PPD), typically limited to the lower extremities; however, these conditions can be distinguished from PMF by characteristic lesion distribution, disease course, and response to standard anti-inflammatory therapies [50,75]. Other rarer clinical entities that can present with poikiloderma but can be excluded solely on anamnestic data or additional characteristic clinical features include chronic graft-versus-host disease (cGVHD), Rothmund–Thomson syndrome, and chronic radiation dermatitis [76,77,78].

PMF is distinguished from CMF on HP by epidermal atrophy, prominent dilated vessels within the papillary dermis, melanin incontinence with dermal melanophages, and a band-like lymphocytic infiltrate, while other features remain relatively similar to those in CMF. Although most cases display the conventional CD3+/CD4+/CD8−/CD7− immunophenotype, some studies have demonstrated a higher prevalence of a CD8+/CD4− cytotoxic phenotype (approximately 38%) compared with CMF (8–23%) [79,80,81].

PMF is characterized by persistent, slowly progressive poikiloderma affecting non–sun-exposed areas, such as the trunk, gluteal regions, and major flexure areas. Its diagnosis is supported by the absence of systemic features, characteristic epidermotropism, pigment incontinence, and dilated dermal vessels on HP, as well as detection of T-cell clonality, which differentiates it from photosensitive dermatoses and other inflammatory mimickers.

## 7. Other Rare Forms of MF

According to the latest WHO-EORTC classification, in addition to FMF, two other rare variants are formally recognized: granulomatous slack skin (GSS) and pagetoid reticulosis (Woringer–Kolopp disease) [1].

GSS is an extremely rare MF variant that presents with loose, sagging skin folds resembling *cutis laxa*, typically localized in the flexural regions, especially the axillary and inguinal areas. Originally described by Convit et al. in 1973 as a granulomatous dermatosis in an adolescent who subsequently died of Hodgkin lymphoma 20 years later, the condition was later analyzed by Ackerman and LeBoit and ultimately recognized as a distinct variant of CTCL [82]. Most often, it affects males in their 3rd and 4th decade of life, and the pathognomonic clinical feature is the progressive development of hanging skin with elastolysis [83,84,85].

GSS’ closest clinical mimic is granulomatous MF (GMF), which shares overlapping HP features but differs clinically in that it presents as patches, plaques, or tumors without a predilection for flexural areas and without the characteristic pendulous skin folds. Other clinical mimics include acquired *cutis laxa* and anetoderma, which produces focal areas of skin laxity with elastic fiber loss but typically manifests as small, well-circumscribed depressions or herniated plaques rather than bulky pendulous folds [86].

The hallmark finding on HP is pronounced elastolysis, typically affecting the full thickness of the dermis, though its extent varies, with partial or segmental involvement possible. This feature distinguishes GSS from GMF, which generally lacks confluent, full-thickness destruction of elastic fibers. Giant cells are evenly distributed within the infiltrate, and the granulomatous component may predominate, sometimes masking the underlying lymphomatous process [84]. In GSS, the neoplastic lymphocytes most commonly exhibit a CD3+/CD4+/CD8− phenotype on IHC, with rare CD8+ variants reported [87]. Some authors propose that observed differences between GMF and GSS are driven by lesion localization and the extent of elastolysis, rather than by a true clinicopathologic divergence between these two entities [84,85].

The significance of GMF and GSS lies in the data reported by some authors that these patients seem more prone to developing other malignancies, such as Hodgkin’s lymphoma and anaplastic large T-cell lymphoma [55,85,88,89].

The indolent course of GSS and lack of subjective symptoms often contribute to delayed diagnosis [82]. Clinicians should maintain a high index of suspicion for associated lymphoproliferative malignancies in patients with GSS and the possibility of extracutaneous spread in patients with GMF ensuring appropriate long-term surveillance [55].

Pagetoid reticulosis (PR) is a localized, indolent MF variant with a good prognosis that typically presents as a solitary, well-demarcated psoriasiform and/or hyperkeratotic patch or plaque on acral sites [90]. The disease was initially described by Woringer and Kolopp in 1939, and the term “pagetoid reticulosis” was introduced in 1973 by Braun-Falco et al. [91]. This term derives from the distinctive HP pattern of atypical lymphocytes scattered throughout the epidermis, resembling the pattern seen in Paget’s disease [92]. In a comprehensive systematic review, the median age at presentation was 47 years, with a slight male predominance observed across reported cases. The same study documented a substantial diagnostic delay, with a mean duration of roughly 8 years between the initial eruption and confirmed diagnosis, likely attributable to minimal symptomatology [90].

Distinguishing PR from CMF patch/plaque stage is important and requires HP confirmation, as clinical features alone are insufficient for definitive diagnosis. Accurate differentiation is essential because localized PR demonstrates excellent 5-year disease-specific survival, whereas CMF prognosis is stage-dependent, with a potential for progression [90]. Other mimickers include psoriasis, atopic dermatitis, chronic eczema, and Bowen’s disease [92].

The hallmark HP finding is striking epidermotropism with atypical lymphocytes dispersed in a pronounced pagetoid pattern involving all layers of a hyperplastic epidermis, while the dermis remains largely spared [55]. On IHC, PR is characterized by phenotypic heterogeneity, most commonly expressing a CD8+ cytotoxic/suppressor profile rather than the CD4+ T-helper phenotype, with occasional CD4-/CD8- double-negative cases reported [90,93].

The disease can also have a more generalized form with a more aggressive course, historically known as Ketron–Goodman disease, but is now reclassified as primary cutaneous aggressive epidermotropic CD8+ T-cell lymphoma (PCAETCL) [94].

Although the solitary, indolent nature of PR may be masked by clinical resemblance to inflammatory dermatoses, clinicians should remain aware of this entity while ensuring that aggressive CD8+ T-cell lymphomas are excluded through careful histopathologic and immunohistochemical evaluation.

Syringotropic mycosis fungoides (STMF) is a very rare adnexotropic variant of CTCL characterized by infiltration of atypical lymphocytes into eccrine sweat glands. Based on the available literature, Sarkany first described this condition in 1969 as syringolymphoid hyperplasia with alopecia (SLHA), and it was later formally recognized as a syringotropic variant of CTCL by Burg and Shmockel in 1992 [95]. The disease is usually asymptomatic, shows a male predominance, and often presents in the 6th decade of life, but can be observed in younger and older patients as well. STMF presents as erythematous patches or plaques with a distinctive punctate appearance, frequently accompanied by alopecia and marked palmoplantar involvement. Less common manifestations include ulcerated, comedo-like, livedoid, and dyshidrosiform lesions [96,97].

Prognostically, STMF demonstrates a significantly more favorable course than FMF, with 5-year disease-specific survival of 100% versus 74% [96]. Other differential diagnoses include syringotropic lichen planus, chronic eczema, and psoriasis. The condition previously known as unilateral laterothoracic exanthem, now referred to as asymmetric periflexural exanthem of childhood (APEC), may have some clinical and HP similarities with STMF. However, APEC occurs almost exclusively in children, making pediatric age a practical exclusion criterion for STMF [95,98].

On HP, STMF is characterized by syringotropism—dense lymphoid infiltrate that surrounds and invades eccrine glands, with characteristic syringometaplasia. On IHC, STMF shares the typical CD3+/CD4+/CD8− T-helper phenotype with frequent CD7 loss, and diagnosis relies on the distinctive histopathologic pattern rather than immunophenotype [95,97].

When patients present with anhidrosis, milia-like papules, and/or alopecia—particularly when accompanied by palmoplantar involvement—and conventional diagnoses fail to explain the clinical presentation, clinicians should consider STMF and obtain deep biopsies that reach the eccrine glands to capture the characteristic adnexotropic infiltrate [95,96,97].

Beyond previously described well-characterized forms, MF may present in numerous additional rare clinical variants that pose a substantial diagnostic challenge. Early recognition of these atypical presentations is important, as misdiagnosis can lead to diagnostic delays lasting years, particularly when these variants mimic benign inflammatory dermatoses. A broad spectrum of clinicopathologic subtypes has been described, including hyperpigmented, papular, purpuric, pustular, hyperkeratotic/verrucous, and psoriasiform MF [85,99,100,101].

## 8. Management and Future Therapeutic Strategies

The therapeutic approach for all MF variants follows the fundamental principle that treatment selection is primarily guided by disease stage rather than clinical and histopathologic subtype [102]. The patients with early-stage (IA-IIA) disease should primarily be treated with SDTs (topical corticosteroids, chlormethine, phototherapy) [102]. Systemic therapies (retinoids, methotrexate, interferons) may be added in refractory cutaneous cases. Advanced-stage disease requires systemic agents, often in combination with SDTs, to control symptoms and reduce tumor burden [102].

BMF, while lacking variant-specific treatment recommendations, may require earlier consideration of systemic therapy given that vesiculobullous transformation can indicate more aggressive disease behavior [16]. IMF, HMF, and PMF typically demonstrate excellent response to phototherapy, particularly with PUVA [103]. The Dutch Cutaneous Lymphoma Group proposed an algorithm for treating FMF, in which, for early-stage disease, they recommend high-potency corticosteroids, PUVA, and low-dose local radiotherapy for solitary lesions or localized plaques [104]. For advanced-stage FMF, standard SDTs alone are often insufficient, necessitating more aggressive approaches including radiotherapy-based modalities and/or systemic therapies—either alone or in combination in order to achieve durable response [104]. The presence of mucin in FMF is often responsible for resistance to SDTs in these patients. Localization of the lymphoid infiltrate in STMF can also make this distinct variant less responsive to SDTs [96]. In a systematic review of 143 cases of PR, localized radiotherapy has shown the highest cure rates and the lowest recurrence [90]. No definitive standard therapy has been established for GSS due to its extreme rarity; however, the Cutaneous Lymphoma French Study Group concluded that while managing GSS can be difficult, methotrexate demonstrated the best efficacy among evaluated treatments [88,105].

For all MF variants, the therapy goals should be individualized to achieve an adequate response, reduce symptoms, and minimize the risk of progression. Allogeneic stem cell transplantation (alloSCT) remains the only potentially curative option for advanced disease [102,106]. Newer agents, including histone deacetylase inhibitors (vorinostat and romidepsin; primarily FDA-approved), brentuximab vedotin (for CD30+ MF), mogamulizumab, alemtuzumab, and denileukin diftitox (DD)-cxdl, fundamentally transformed the treatment paradigm for MF [102,106,107,108]. The use of these drugs has expanded the spectrum of treatment options for relapsed/refractory disease, though their specific efficacy across MF variants remains to be determined [102,107,108,109].

## 9. Discussion

MF presents a complex diagnostic challenge in dermatology. It is often regarded as one of the discipline’s most notorious “great imitators”. In its early stages, MF poses a significant diagnostic challenge, even when presenting with classical clinical features; this difficulty is further compounded by rare or atypical variants that closely mimic benign inflammatory dermatoses. The patient journey from the onset of early MF symptoms to diagnosis often involves repeated cycles of incorrect clinical and/or HP diagnoses and ineffective therapies [10]. The documented diagnostic delay for MF is significant, with median intervals ranging from 3 to 7 years between the onset of symptoms and a definitive diagnosis, extending up to 12 years in specific cases [53,110,111]. This highlights the urgent need for increased clinical vigilance, especially when assessing chronic, treatment-resistant skin conditions.

The remarkable clinical heterogeneity of MF reflects profound biological complexity operating at genomic, clonal, microenvironmental, and microbial levels. Integrated genomic analyses have identified at least 86 putative driver genes affecting multiple signaling pathways, with no consistent pattern of clonal driver mutations across patients, meaning different individuals develop MF through fundamentally different molecular mechanisms [13]. Beyond genomic variation, MF exhibits polyclonal architecture, with multiple neoplastic T-cell clones (median 11 clones, range 2–80 per sample) continuously seeding the skin via hematogenous circulation, creating polyclonal lesions with considerable variation between patients and even between different anatomic sites in the same individual [12]. The tumor microenvironment (TME) in MF comprises the complex ecosystem of non-malignant cells, cytokines, chemokines, extracellular matrix components, and microbial factors that surround and interact with malignant T-cells in the skin [112]. This microenvironment is not merely a passive bystander but actively shapes disease pathogenesis, progression, and therapeutic resistance. Spatial transcriptomics reveals that folliculotropic variants exhibit highly inflammatory phenotypes with enhanced metabolic activity, which explains their more aggressive behavior [113]. Microbial influences add another layer of complexity: pathogenic *Staphylococcus aureus* strains carrying virulence factors activate NF-κB and IL-1β signaling, fueling disease progression, and multi-omics data integration suggests that microbial influence accounts for much of the inter-patient transcriptional heterogeneity [114,115]. This multilayered biological complexity explains why MF has such variable clinical manifestations. The absence of a single unifying molecular signature means there is no pathognomonic feature that distinguishes MF from inflammatory conditions. This biological diversity fundamentally justifies the MF designation as dermatology’s “great imitator” and necessitates consideration of differential diagnoses exceeding 50 conditions, reflecting its exceptional capacity for clinical mimicry [32].

Beyond the diagnostic complexity posed by MF mimicking numerous inflammatory conditions, clinicians must remain vigilant for an additional layer of diagnostic challenge: patients with MF are at increased risk for diagnosis of second primary hematologic and solid organ malignancies. In the comprehensive surveillance, epidemiology, and end results-18 (SEER-18) analysis of 6,742 MF patients, 7.5% developed second primary malignancy [116]. An especially elevated risk was identified for hematologic malignancies, representing 36% of second primary cancers, as well as for solid tumors, comprising 64% [116]. Hodgkin lymphoma, non-Hodgkin lymphoma, and melanoma were the most frequently observed second malignancies, with additional elevated risks reported for lung, bladder, breast (in females), prostate, colorectal, and renal cancers [116]. A Dutch nationwide study of 1,024 MF patients found that 29% developed at least one other primary malignancy during a mean 10-year follow-up, with significantly elevated risks for cutaneous and hematologic malignancies [117]. These findings underscore the importance of comprehensive cancer surveillance in MF patients, including regular lymph node examinations, pigmented-lesion-focused skin examinations, and ensuring that age- and sex-appropriate cancer screenings are performed in accordance with up-to-date recommendations.

These diagnostic challenges highlight a fundamental limitation of current classification systems, which rely predominantly on clinicopathologic features while inadequately integrating the molecular and immunological biomarkers that increasingly influence prognosis and treatment selection. The 2018 WHO-EORTC classification and the 2022 5th edition of the WHO classification of hematolymphoid tumors (WHO-HAEM5) recognize only three MF variants: FMF, PR, and GSS [1,118]. However, the International Consensus Classification (ICC) of mature lymphoid, histiocytic, and dendritic cell tumors excluded subtypes of MF from their latest classification in 2022 [119]. The inclusion of additional clinical variants of MF in the existing formal classification systems remains a matter of ongoing debate. It underscores the need to balance clinical applicability, biological insight, and principles of disease classification.

Expanding MF classification to include additional variants is supported by data demonstrating clinically meaningful differences in prognosis, treatment response, and diagnostic delay patterns between distinct clinicopathological types of MF [9,56]. However, one must keep in mind that different clinical presentations do not necessarily reflect different tumor biology. Many of the described variants primarily facilitate diagnosis by highlighting characteristic clinical morphologies or patterns of distribution, such as HMF and PMF [85]. Based on their prognosis and treatment response, current knowledge indicates that they do not require recognition as separate MF subtypes in formal classification systems. Current staging systems and guideline recommendations emphasize that disease stage, folliculotropism, large-cell transformation, age over 60 years old, and male gender convey the most robust prognostic information, allowing clinically relevant distinctions without excessive expansion of classification categories [120].

Future classifications should focus on integrating molecular data to determine which clinical variants truly represent distinct biological entities and which represent only phenotypic diversity [121]. Creating a globally accepted classification system would provide consistent, equivalent diagnoses for both clinical and scientific purposes. However, no classification can fully reflect the heterogeneity of MF; yet selective, evidence-based, and future molecularly driven analyses would enable the identification of key subtypes, improve prognostic accuracy, and guide tailored treatment strategies. Therefore, excluding MF variants from the ICC may be an overcorrection that sacrifices clinically useful information for the sake of classification simplicity.

Throughout our years of clinical practice, we have encountered MF in its many disguises—hypopigmented patches mistaken for vitiligo, ichthyosiform plaques mistaken for chronic eczema, bullous lesions attributed to autoimmune blistering disorders, poikilodermatous changes mistaken for connective tissue disease, and folliculotropic variants masquerading as acneiform eruptions or scarring alopecia. Each misdiagnosis, diagnostic delay, and each patient who endured years of ineffective treatments before receiving the correct diagnosis has reinforced a fundamental clinical truth: MF is perhaps dermatology’s “greatest imitator”, rivaling and arguably surpassing even syphilis in its capacity to mimic benign inflammatory dermatoses.

This review focuses on rare MF clinical variants, as recognition of these atypical presentations is essential to reduce diagnostic delays that negatively affect patient outcomes and quality of life. The clinical variability associated with MF requires a thorough diagnostic approach that includes detailed clinical examination and dermoscopic evaluation. Dermoscopy has proven to be highly useful in identifying characteristic patterns—such as fine short linear and/or spermatozoa-like blood vessels, fine and patchy scaling, and perifollicular accentuation—that help distinguish MF from its inflammatory mimickers with high specificity [38]. However, one must keep in mind that many dermoscopic finding are non-specific and confirming the diagnosis requires HP evaluation.

By summarizing the unique cutaneous signs and dermoscopic features of these rare MF variants, as well as their most common mimickers, this narrative review aims to provide clinicians with practical diagnostic clues (Table 1). These clinical manifestations should aid in earlier recognition, guide appropriate biopsy decisions, and ultimately prevent the use of potentially harmful treatments for misdiagnosed inflammatory conditions. The persistent emphasis on differential diagnosis throughout this work reinforces the fundamental clinical principle that MF must remain in the differential diagnosis of any chronic, treatment-resistant dermatosis, particularly when clinical features deviate from typical inflammatory patterns or when lesions continue to progress despite appropriate therapy.

We wrote this review because we believe that MF, despite its rarity and complexity, can be diagnosed earlier when clinicians remain vigilant to its variable manifestations. The rare variants we have described—BMF, IMF, HMF, FMF, and PMF—are not merely academic curiosities; they are real clinical entities that we have encountered, biopsied, diagnosed, and treated. By sharing the distinctive cutaneous signs and dermoscopic features that have guided our diagnostic decisions, we hope to equip colleagues with practical tools that facilitate earlier recognition and reduce the diagnostic delays that continue to characterize this disease. In addition, we have addressed other uncommon variants, including GSS, GMF, PR, and STMF, to provide a broader clinical context and to reflect the full spectrum of rare MF variants.

## 10. Conclusions

MF will continue to pose a significant diagnostic challenge by mimicking benign inflammatory dermatoses and evading early recognition. Nevertheless, heightened clinical awareness, systematic use of dermoscopy, a histopathology-based diagnostic approach, appropriate integration of molecular diagnostics when available, and comprehensive long-term follow-up may facilitate earlier diagnosis, enable more effective therapeutic intervention, and ultimately improve patient outcomes. In this context, maintaining dual vigilance is essential: clinicians must not only recognize MF among its numerous mimickers but also remain alert to the risk of second primary malignancy throughout the patient’s lifetime. Looking ahead, the future of MF care lies in integrating molecular insights into clinical practice with the development of classification systems that incorporate tumor biology and molecular signatures alongside traditional morphologic criteria.

## Figures and Tables

**Table 1:** Summary of clinical and dermoscopic findings, differential diagnoses, and prognosis in rare mycosis fungoides variants.

MF Variant	Key Clinical Features	Dermoscopic Clues *	Most Common Clinical Differential Diagnoses	Prognosis
**Bullous**	Vesicles/bullae overlying patches/plaques; trunk and extremities	Liner vessels, whitish scale, components of blister formation/resolution, ulceration	Bullous pemphigoid, pemphigus, linear IgA dermatosis, bullous drug eruption, infectious diseases, Hailey-Hailey disease	Poor
**Ichthyosiform**	Generalized scaling resembling ichthyosis; dry, scaly plaques; alone or with classic/follicular MF; trunk and extremities	White geometric scales, linear/spermatozoa vessels, and orange structureless areas	*Ichthyosis vulgaris*, acquired ichthyosis, chronic eczema, xerosis in chronic renal disease, and endocrinologic disorders	Favorable
**Hypopigmented**	Hypopigmented patches/plaques; trunk and limbs; most common in children	Linear vessels, spermatozoa-like vessels, orange-yellow areas, and white scales in furrows	Vitiligo, pityriasis alba, post-inflammatory hypopigmentation, lichen sclerosus	Favorable
**Folliculotropic (early-stage** **disease)**	Follicular papules, keratosis pilaris-like lesions, and/or acneiform lesions; face/trunk/limbs; pruritus	Dilated follicles, follicular plugs, lack of hairs, perifollicular accentuation, spermatozoa-like vessels, yellow dots	Acne, rosacea, keratosis pilaris, alopecia areata, folliculitis	Favorable
**Folliculotropic** **(advanced-stage disease)**	Infiltrated plaques/tumors, alopecia; head and neck preference; dense perifollicular infiltrates	Dotted dilated vessels, broken dystrophic hairs, spermatozoa-like vessels, and absence of follicular dots	Deep fungal infection, furunculosis, *hidradenitis suppurativa*, sarcoidosis	Poor
**Poikilodermatous**	Atrophy, telangiectasia, hyper and hypopigmentation; reticulated pattern; flexural areas and trunk	Focal white and brown structureless areas, white patchy scales, brown reticular lines, polymorphous vessels	Dermatomyositis, radiodermatitis, discoid lupus erythematosus, cGVHD	Favorable
**Granulomatous**	Patches, plaques with irregular areas of induration or tumors; trunk and distal parts of extremities	Lack of studies	GSS, CMF, sarcoidosis, granuloma annulare, interstitial granulomatous dermatitis, infectious granulomatous dermatoses	Intermediate to unfavorable **
**Granulomatous slack skin**	Bulky, pendulous skinfolds usually located in flexural areas	Lack of studies	GMF, acquired *cutix laxa*, anetoderma	Intermediate to relatively favorable ***
**Pagetoid** **reticulosis**	Solitary, well-demarcated psoriasiform and/or hyperkeratotic patch or plaque; acral sites	Lack of studies	CMF, psoriasis, atopic dermatitis, chronic eczema, Bowen’s disease	Favorable
**Syringotropic**	Erythematous patches or plaques sometimes exhibiting a punctate appearance, alopecia; palmoplantar involvement	Lack of studies	FMF, syringotropic lichen planus, chronic eczema, psoriasis	Favorable

Abbreviations: MF—mycosis fungoides; cGVHD—chronic graft-versus-host-disease; GSS—granulomatous slack skin; CMF—classic mycosis fungoides; FMF—folliculotropic mycosis fungoides; * The dermoscopic features were obtained from previously published literature and are appropriately referenced throughout the manuscript. Dermoscopic patterns in MF are mostly non-specific, although perifollicular accentuation and spermatozoa-like vessels represent relatively specific clues that may support the diagnosis. ** Possible extracutaneous spread; *** Indolent, but therapy-resistant course; uncertain long-term outcome due to the risk of associated malignancies.

## Data Availability

No new data were created or analyzed in this study.

## Abbreviations

The following abbreviations are used in this manuscript:

MF	mycosis fungoides
pCTCL	primary cutaneous T-cell lymphoma
HP	histopathology/histopathological
IHC	immunohistochemistry/immunohistochemically
CMF	classic mycosis fungoides
SS	Sézary syndrome
BMF	bullous mycosis fungoides
Th2	T-helper type 2
IL-5	interleukin-5
IL-10	interleukin-10
IFN-γ	interferon-γ
IMF	ichthyosiform mycosis fungoides
STD	skin-directed therapy
AI	acquired ichthyosis
ATLL	adult T-cell leukemia/lymphoma
HTLV-1	human T-cell leukemia virus type
HMF	hypopigmented mycosis fungoides
PLC	pityriasis lichenoides chronica
FMF	folliculotropic mycosis fungoides
WHO-EORTC	World Health Organization—European Organization on Research and Treatment of Cancer
PMF	poikilodermatous mycosis fungoides
DLE	discoid lupus erythematosus
PPD	pigmented purpuric dermatosis
cGVHD	chronic graft-versus-host disease
GSS	granulomatous slack skin
GMF	granulomatous mycosis fungoides
PR	pagetoid reticulosis
PCAETCL	primary cutaneous aggressive epidermotropic CD8+ T-cell lymphoma
STMF	syringotropic mycosis fungoides
SLHA	syringolymphoid hyperplasia with alopecia
APEC	asymmetric periflexural exanthem of childhood
TME	tumor microenvironment
SEER-18 analysis	surveillance, epidemiology, and end results-18 analysis
WHO-HAEM5	5th edition of the World Health Organization classification of hematolymphoid tumors
ICC	international consensus classification
PUVA	psoralen + ultraviolet A(radiation)
AlloSCT	allogeneic stem cell transplantation
FDA	the Food and Drug Administration
